# Concentric and Eccentric Remodelling of the Left Ventricle and Its Association to Function in the Male Athletes Heart: An Exploratory Study

**DOI:** 10.3390/jcdd10070269

**Published:** 2023-06-23

**Authors:** Christopher Johnson, Nicholas Sculthorpe, Keith George, Martin Stout, William Procter, Robert M. Cooper, David Oxborough

**Affiliations:** 1 Research Institute for Sport and Exercise Sciences, Liverpool John Moores University, Liverpool L3 3AF, UK; 2 Sport and Physical Activity Institute, University of the West of Scotland, Glasgow G72 0LH, UK; 3 School of Healthcare Science, Manchester Metropolitan University, Manchester M15 6BH, UK; 4 Department of Cardiology, Barts Heart Centre, London EC1A 7BE, UK

**Keywords:** athletes heart, left ventricle, echocardiography, speckle tracking echocardiography, strain imaging

## Abstract

Aims: To compare (1) conventional left ventricular (LV) functional parameters, (2) LV peak strain and strain rate and (3) LV temporal strain and strain rate curves in age, ethnicity and sport-matched athletes with concentric, eccentric and normal LV geometry. Methods: Forty-five male athletes were categorised according to LV geometry including concentric remodelling/hypertrophy (CON), eccentric hypertrophy (ECC) or normal (NORM). Athletes were evaluated using conventional echocardiography and myocardial speck tracking, allowing the assessment of myocardial strain and strain rate; as well as twist mechanics. Results: Concentric remodelling was associated with an increased ejection fraction (EF) compared to normal geometry athletes (64% (48–78%) and 56% (50–65%), respectively; *p* < 0.04). No differences in peak myocardial strain or strain rate were present between LV geometry groups including global longitudinal strain (GLS; CON −16.9% (−14.9–20.6%); ECC −17.9% (−13.0–22.1%); NORM −16.9% (−12.8–19.4%)), global circumferential strain (GCS; CON −18.1% (−13.5–24.5%); ECC −18.7% (−15.6–22.4%); NORM −18.0% (−13.5–19.7%)), global radial strain (GRS; CON 42.2% (30.3–70.5%); ECC 50.0% (39.2–60.0%); NORM 40.6 (29.9–57.0%)) and twist (CON 14.9° (3.7–25.3°); ECC 12.5° (6.3–20.8°); NORM 13.2° (8.8–24.2°)). Concentric and eccentric remodelling was associated with alterations in temporal myocardial strain and strain rate as compared to normal geometry athletes. Conclusion: Physiological concentric and eccentric remodelling in the athletes heart is generally associated with normal LV function; with concentric remodelling associated with an increased EF. Physiological concentric and eccentric remodelling in the athletes heart has no effect on peak myocardial strain but superior deformation and untwisting is unmasked when assessing the temporal distribution.

## 1. Introduction

The ‘athletes heart’ (AH) is a term used to describe the complex physiological adaptation in structure, function and electrical remodelling of the heart that occurs in response to chronic exercise [1,2]. The phenotypic expression of adaptation is heterogenous and dependent upon factors including sex, age, ethnicity, body size, sporting discipline and training volume [3]. Although altered left ventricular (LV) geometry in athletes is a widely established response to exercise stimulus [4,5], concentric remodelling/hypertrophy is often recognised as a rare manifestation [4,5,6,7,8,9]. In view of this, the presence of concentric hypertrophy/remodelling often warrants further investigation [3] to differentiate from inherited cardiac conditions (ICC) that may predispose an athlete to sudden cardiac death (SCD) such as hypertrophic cardiomyopathy (HCM) [10,11,12]. Conversely, eccentric hypertrophy is a common manifestation of the AH and is associated with high training volumes [4,6]. The enlargement of the LV volume presents with a diagnostic dilemma from the differentiation of dilated cardiomyopathy (DCM) [10,11,13] and, therefore, an accurate functional assessment is important.

Echocardiography plays an important role in the assessment of LV structure and function when differentiating AH from cardiomyopathy. Ejection fraction (EF) is the most widely utilised measure of LV systolic function. A reduced EF in the presence of eccentric hypertrophy was previously identified in athletes [14]; exacerbating the differentiation of physiological eccentric hypertrophy in athletes from DCM. Furthermore, a preserved/increased EF in the presence of concentric hypertrophy is routinely observed in HCM patients [15]. Increasingly, studies suggest that novel techniques of two-dimensional speckle tracking echocardiography (2DSTE) and global/temporal strain may provide additional diagnostic value [11,16,17,18].

LV global longitudinal strain (GLS) is a measure of systolic function and is the most commonly cited cardiac deformation parameter in clinical disease and AH populations [11]. A recent meta-analysis concluded that GLS in athletes is comparable to that of matched healthy controls, regardless of sporting discipline [19], whereas there are data demonstrating reduced GLS in patients with HCM and DCM. [13,20,21]. The assessment of more detailed LV mechanics including global circumferential, radial strain (GCS and GRS, respectively) and twist are generally comparable between athletes and healthy matched controls [19] but may be reduced in HCM and DCM compared to athletes and healthy controls [22,23]. That aside, there is heterogeneity between studies which may, in part, be related to different LV geometry and or variable disease phenotypes. Many of the studies present only the peak values of strain and twist; however, detailed mechanics across the cardiac cycle are obtainable and, therefore, these data may be valuable in detecting subtle differences between athletes of variable LV geometry.

In view of this, the aims of the current study were to compare (1) conventional LV functional parameters, (2) LV peak strain and strain rate and (3) LV temporal strain and strain rate curves in age, ethnicity and sport-matched athletes with concentric, eccentric and normal LV geometry.

## 2. Materials and Methods

### 2.1. Participants

Forty-five elite male athletes of various sporting disciplines were recruited through the Athlete Screening Programme at Liverpool John Moores University. Participants were aged between 18 and 35 years of age. The athletes competed at various levels ranging from club to international level. All athletes had a negative family history and were asymptomatic. All participants were free from known hypertension, valve disease, ischaemic heart disease, diabetes, respiratory, endocrinal, liver or renal disease. Participants were not taking cardiovascular medication. All participants provided written informed consent prior to the investigation, and the study was granted ethical approval by the Ethics Committee of Liverpool John Moores University.

### 2.2. Design

This study utilised a cross-sectional design with athletes grouped according to LV geometry as concentric remodelling/hypertrophy (CON; *n* = 15), eccentric hypertrophy (ECC; *n* = 15) or normal (NORM; *n* = 15), in adherence to the established British Society of Echocardiography (BSE) reference values [24]. Participants completed a comprehensive cardiovascular assessment, including a 12-lead electrocardiogram (ECG) and a two-dimensional transthoracic echocardiogram (TTE) with subsequent 2DSTE. All athlete data were collected during routine pre-participation screenings as advised by the sporting governing bodies and was overseen by a specialist sports cardiologist.

### 2.3. Procedures

#### 2.3.1. Anthropometric and Electrocardiography

Height and weight were recorded using standard scales (SECA stadiometer, SECA, Germany and SECA scale, SECA, Hamburg, Germany, respectively). A standard 12-lead ECG (Cardiovit MS-2010, Schiller, Baar, Switzerland) was taken following routine procedure to identify training-related and training-unrelated ECG changes [25]. 

#### 2.3.2. Echocardiography

Resting echocardiography was performed by a single clinically accredited echocardiographer (D.O). Images were acquired using a commercially available ultrasound system (E95, GE Healthcare, Oslo, Norway) and a 1.5–4 MHz phased array transducer. Echocardiographic images were acquired with the participant in the left lateral decubitus position. Full minimum data were acquired [26], with each image recorded over a minimum of 3 cardiac cycles. Images were stored in a raw DICOM format and exported to an offline workstation (EchoPac Version 203, GE Healthcare, Norway) for subsequent conventional and 2DSTE analysis. Analysis was completed by a second sonographer (C.J). Data were collected and managed using REDCap electronic data capture tool [27].

#### 2.3.3. Conventional Echocardiography

Two-dimensional, Doppler and tissue Doppler (TDI) LV structural and functional measurements were made according to the current BSE guidelines [24,26]. LV internal dimensions at end-diastole and end-systole (LVIDd and LVIDs, respectively), interventricular septal thickness and LV posterior wall thickness at end-diastole (IVSd and PWTd, respectively) allowed the calculation of LV mass using the formula [0.8 (1.04 (LVIDd + IVSd + PWTd)^3^ LVIDd^3^) + 0.6]. To provide a comprehensive assessment of LV mean wall thickness (MeanWT), eight measurements were averaged from a parasternal short axis orientation at basal and mid-levels from the anteroseptum, infero-septum, posterior wall and lateral wall; with the maximum wall thickness (MaxWT) also reported. Relative wall thickness (RWT) was calculated using the formula [(RWT = IVSWTd + PWTd)/LVIDd]. LV end-diastolic volume (LVEDV), LV end systolic volume (LVESV), stroke volume (SV), cardiac output (CO) and ejection fraction (EF) were calculated using the Simpson’s biplane method. Pulsed-wave tissue Doppler imaging (TDI) assessed the septum and lateral wall for systolic (S′), early (E′) and late (A′) diastolic velocities. Spectral Doppler provided mitral valve inflow early diastolic and late diastolic velocities (MV E and MV A, respectively), LV outflow tract (LVOT) velocity, LVOT velocity time integral (LVOT VTI) and aortic flow velocity [24,26]. 

The combination of indexed LV mass and RWT was used to define geometry according to the BSE guidelines [24]. LV geometry was classified as ‘normal’ if RWT was ≤0.42 in the presence of normal LV mass (≤110 g/m^2^ in males). A normal LV mass with a RWT > 0.42 defines ‘concentric remodelling’. Increased LV mass (>110 g/m^2^ in males) with a RWT ≤ 0.42 classifies ‘eccentric hypertrophy’. The presence of increased LV mass and a RWT > 0.42 represents ‘concentric hypertrophy’. 

All structural indices were scaled allometrically to body surface area (BSA) based on the principles of geometrical similarity, i.e., linear dimensions were scaled to BSA^0.5^, areas directly to BSA, and mass and volumes to BSA^1.5^.

#### 2.3.4. Two-Dimensional Speckle Tracking Echocardiography

Two-dimensional images optimised for 2DSTE were collected to assess peak global myocardial deformation including longitudinal (GLS), circumferential (GCS), radial (GRS) strain and twist; as well as peak systolic, early diastolic and late diastolic global longitudinal (GLSr S, GLSr E and GLSr A), circumferential (GCSr S, GCSr E and GCSr A) and radial (GRSr S, GRSr E and GRSr A) strain rate.

Images were acquired at frame rates between 40 and 90 frames per second (FPS) [28]. Gain settings were optimised and breathe-hold techniques utilised to clearly delineate the endocardial and epicardial borders with image width and depth focused on the LV chamber. A minimum of three cardiac cycles were collected per image with the cardiac cycle providing the optimal endocardial delineation selected for subsequent analysis. The endocardium was manually traced with the region of interest adjusted to incorporate the whole of the myocardium while excluding excess structures such as the papillary muscles and pericardium [29].

Spectral Doppler traces of the MV and aortic valve (AV) provided definition of true LV end-diastolic and end-systolic event timing. LV GLS and GLSr were assessed using the apical four-chamber, two-chamber and three-chamber views, providing a global value based on the average of 18 segments. The parasternal short-axis view allowed the assessment of LV GCS, GCSr, GRS and GRSr. Peak values were averaged from six myocardial segments assessed at the basal level, the level of the mitral valve; whilst the papillary muscle level provided the same data at mid-level. The average GCS, GCSr, GRS and GRSr were calculated from basal and mid values and, hence, 12 myocardial segments. Basal rotation was measured using the circumference of the LV at the mitral valve level. A parasternal short-axis view at the level of the apex, defined as the level just above the point of systolic cavity obliteration, was used to assess apical rotation. LV twist was calculated as the net difference between apical and basal rotation.

#### 2.3.5. Temporal Speckle Tracking Echocardiography

The raw STE strain data were extracted from the GE software and exported to a bespoke Python programme which averaged the data for GLS, GCS, GRS and twist. A cubic spline of the averaged data provided 2000 data points (1000 for systole and 1000 for diastole) to allow the production of strain values at 5% increments of the cardiac cycle (10 systolic and 10 diastolic time points). The temporal data across systole and diastole in 5% increments of absolute cardiac cycle were defined and plotted to create temporal strain curves and graphs.

### 2.4. Statistical Analysis

Continuous data are presented as median (range). Due to the small sample size and non-normally distributed data, a Kruskal–Wallis test was applied to all variables across all three LV geometry groups. Bonferroni post hoc analysis was applied to establish differences between pairwise group comparisons. A *p* value of <0.05 was considered statistically significant. Statistical analyses were performed using IBM SPSS (version 28, SPSS, Chicago, IL, USA).

### 2.5. Laboratory 2DSTE Intra-Observer Reproducibility

On a separate data set, our laboratory demonstrated excellent repeated analysis reproducibility for peak GLS (CoV −3%, ICC 0.85), GCS (CoV −5%, ICC 0.85), GRS (CoV 12%, ICC 0.88) and Twist (CoV 10%, ICC 0.89). Repeated acquisition reproducibility was consistently lower than repeated analysis but still demonstrated good reproducibility for peak GLS (CoV −6%, ICC 0.62) and GCS (CoV −10%, ICC 0.62); with lower reproducibility demonstrated for peak GRS (CoV 21%, ICC 0.55) and Twist (CoV 33%, ICC 0.22).

## 3. Results

### 3.1. Demographics

Baseline demographics for all athlete groups are presented in Table 1. All athlete groups were matched for age, sporting discipline (soccer *n* = 8, cycling *n* = 6 and rugby *n* = 1 per group) and ethnicity (Caucasian *n* = 10, Black/African/Caribbean *n* = 4 and Native Hawaiian/Pacific Islanders *n* = 1 per group). No significant differences were present between groups for training volume, height, body mass, BSA and HR. All athletes presented with a normal ECG according to the International Criteria [25].

### 3.2. Conventional Echocardiography

**Figure 1:**
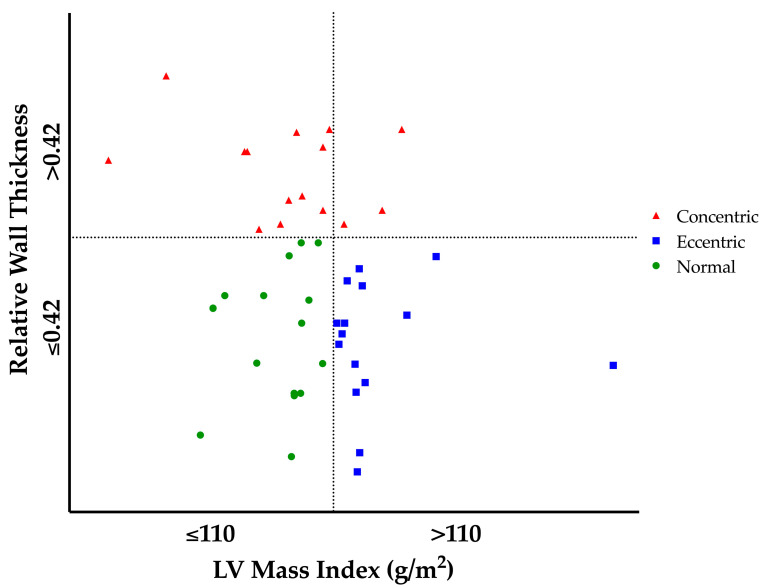
Athlete Left Ventricular Geometry. Upper left quadrant represents athletes with Concentric Remodelling; upper right quadrant represents athletes with Concentric Hypertrophy; lower left quadrant represents athletes with Normal Geometry and lower right quadrant represents athletes with Eccentric Hypertrophy. LV, left ventricular.

**Figure 2:**
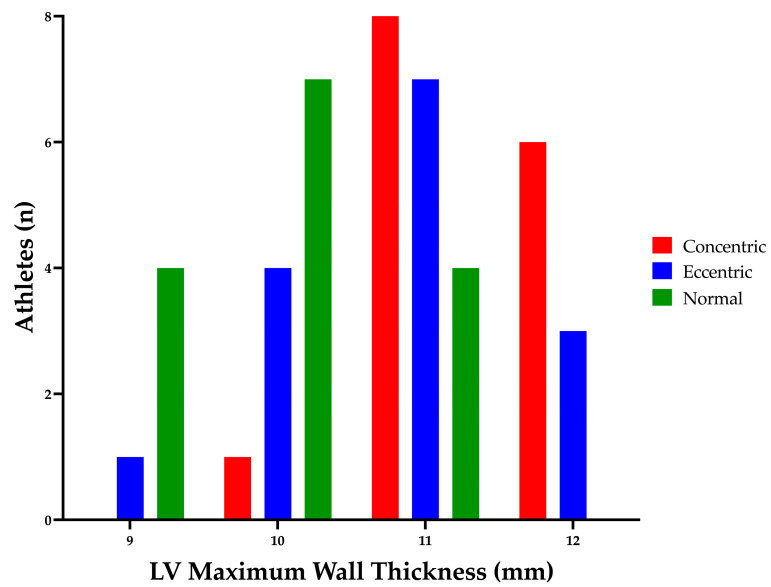
Athlete Left Ventricular Maximum Wall Thickness. LV, left ventricular.

**Figure 3:**
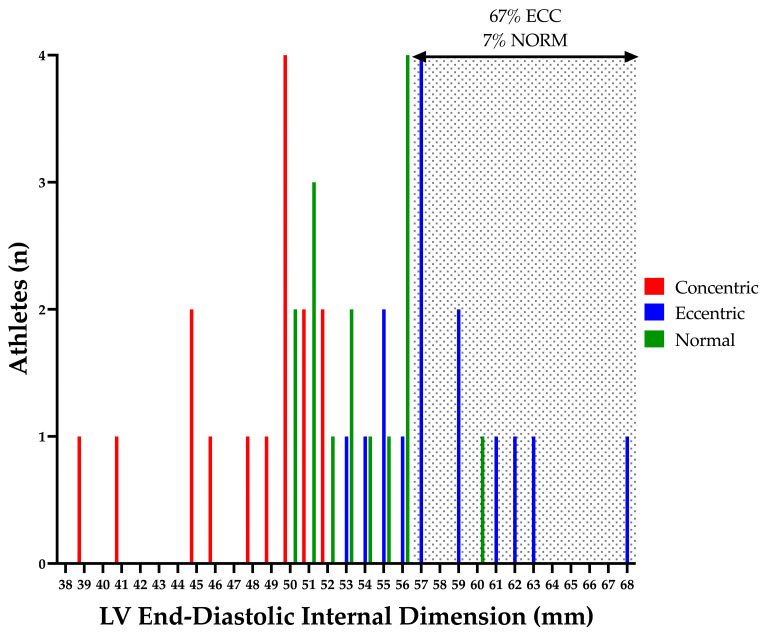
Athlete Left Ventricular End-Diastolic Dimension. Shaded area highlights athletes with a left ventricular end-diastolic internal dimension outside of the normal limits (7% of NORM and 67% of ECC) [26]. ECC, eccentric hypertrophy; LV, left ventricular; NORM, normal geometry.

Structural and functional indices of the LV are presented in Table 2 and Table 3. LV cavity size and mass were different between groups as per geometry selection (see Figure 1). ECC demonstrated increased LV cavity size, wall thickness and mass as compared to NORM; with CON also presenting increased wall thickness with markedly smaller cavity dimensions than NORM (see Figure 2 and Figure 3). CON demonstrated increased RWT compared to both ECC and NORM. No significant difference in LV volumes was observed between groups. EF was significantly higher in CON than NORM (64% (48–78%) and 56% (50–65%), respectively; *p* < 0.04); with no differences in SV or CO between groups. No differences were present in LV TDI and Doppler parameters between groups.

### 3.3. Two-Dimensional Speckle Tracking Echocardiography

LV peak myocardial strain and strain rate parameters are presented in Table 4. There were no significant differences between groups for peak GLS, GCS, GRS and twist. No significant differences were observed between groups for systolic, early diastolic or late diastolic strain rate.

### 3.4. Temporal Speckle Tracking Echocardiography

**Figure 4:**
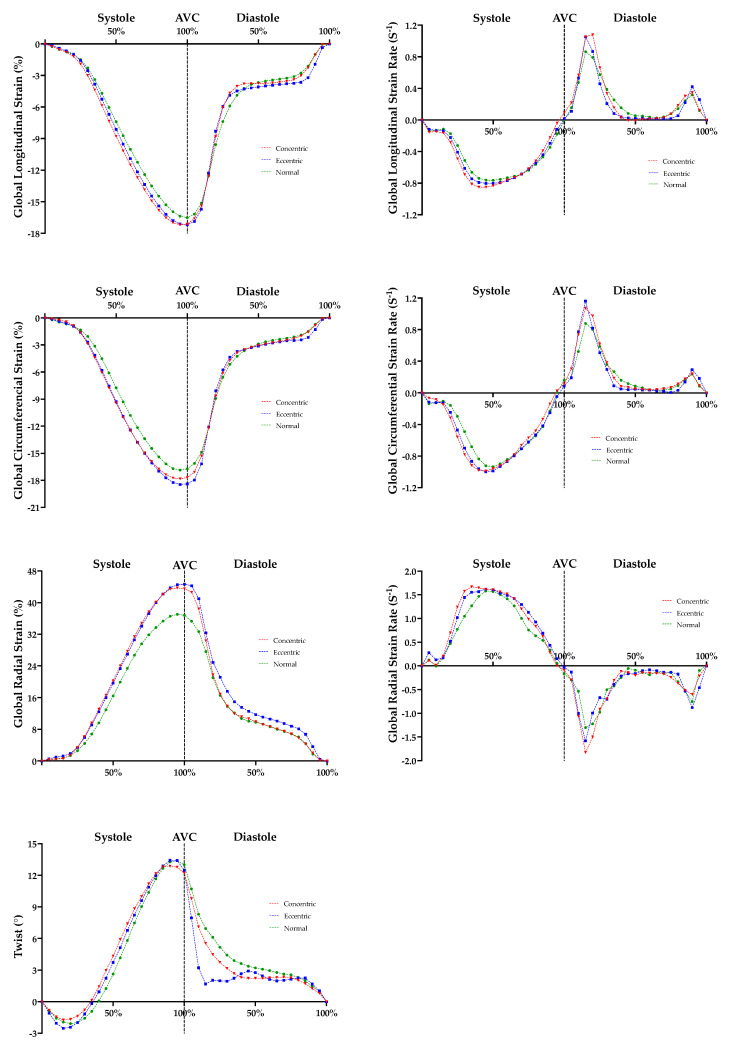
Athlete Left Ventricular Global Longitudinal, Global Circumferential and Global Radial Temporal Strain and Strain Rate, and Twist, Curves. Red shading represents significant difference between concentric and normal. Blue shading represents significant difference between eccentric and normal. AVC, aortic valve closure.

LV temporal myocardial strain and strain rate curves are presented in Figure 4. Early mid systolic longitudinal (40–55% systole; 40% *p* = 0.045; 45% *p* = 0.039; 50% *p* = 0.037; 55% *p* = 0.045) and circumferential (35–50% systole; 35% *p* = 0.025; 40% *p* = 0.015; 45% *p* = 0.018; 50% *p* = 0.032) strain was superior in CON compared to NORM; with increased early mid and decreased late systolic longitudinal (25–40% systole and 85–95% systole; 25% *p* = 0.029; 30% *p* = 0.021; 35% *p* = 0.021; 40% *p* = 0.022; 85% *p* = 0.048; 90% *p* = 0.005; 95% *p* = 0.004), circumferential (20–40% systole; 20% *p* = 0.010; 25% *p* < 0.001; 30% *p* < 0.001; 35% *p* < 0.001; 40% *p* = 0.046) and radial (25–30% systole; 25% *p* = 0.031; 30% *p* = 0.009) strain rate observed in CON compared to NORM. ECC demonstrated superior early mid systolic circumferential (45% systole; 45% *p* = 0.046) strain and increased early mid systolic circumferential (25–35% systole; 25% *p* = 0.029; 30% *p* = 0.008; 35% *p* = 0.011) strain rate compared to NORM; with increased early diastolic radial (5–10% diastole; 5% *p* = 0.047; 10% *p* = 0.029) strain and enhanced untwisting (15–20% diastole; 15% *p* = 0.034; 20% *p* = 0.039) also observed in ECC compared to NORM. No significant temporal myocardial strain or strain rate differences were demonstrated between CON and ECC.

## 4. Discussion

The main findings of the current study were as follows: (1) Physiological concentric and eccentric remodelling in the AH is associated with normal conventional LV functional parameters as measured by TDI and Doppler. Concentric remodelling is associated with an increased EF as compared to normal geometry. (2) Physiological concentric and eccentric remodelling in the AH has no effect on peak myocardial strain and strain rate, but (3) there are temporal differences present with superior early mid systolic longitudinal and circumferential strain and strain rate demonstrated in concentric and eccentric remodelling as compared to normal geometry. Furthermore, eccentric remodelling is associated with an earlier and more marked relaxation/untwisting in early diastole.

### 4.1. Conventional Echocardiography

**Figure 5:**
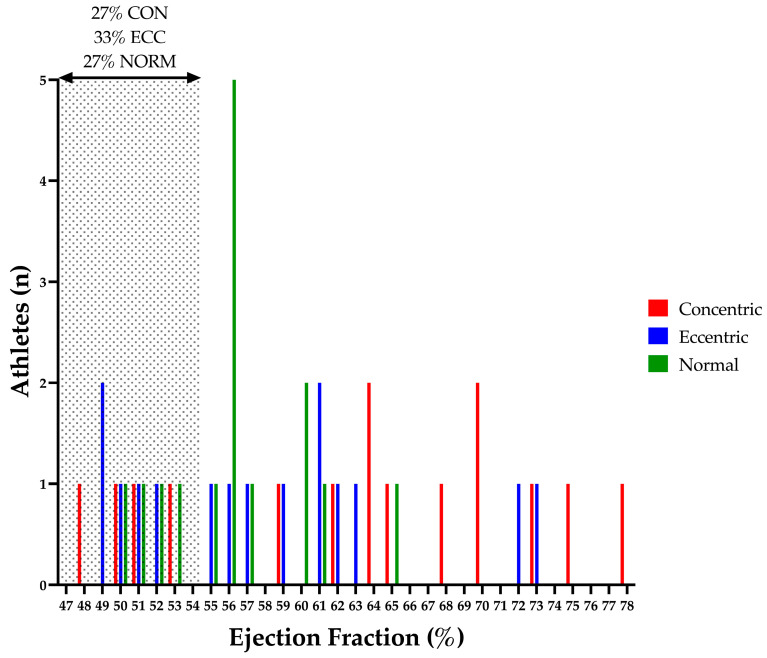
Athlete Left Ventricular Ejection Fraction. Shaded area highlights athletes with a left ventricular ejection fraction outside of the normal limits (27% of NORM, 67% of ECC and 27% CON) [26]. CON, concentric remodelling/hypertrophy; ECC, eccentric hypertrophy; LV, left ventricular; NORM, normal geometry.

In the current study, conventional TDI and Doppler measures of LV systolic and diastolic function were comparable between athletes regardless of LV geometry. The majority of athletes presented with an EF within normal limits; however, an EF below 55% was seen in a similar percentage of athletes from all geometry groups (CON 27%, ECC 33% and NORM 27%; see Figure 5). This reduction in EF within a minority of athletes is widely recognised within the existing literature and consensus documents and is likely attributed to a physiological functional reserve [11]. Although a comparable number of athletes from each group presented with a reduced EF, overall, CON demonstrated a statistically significant increase in EF (see Table 2). Importantly, the increase in EF did not result in a superior SV or CO within this group.

The increased EF observed in athletes with concentric remodelling may be explained by the interactivity of wall thickness and systolic function. A model presented by MacIver and Townsend [30] demonstrated EF was preserved in the presence of systolic impairment (as measured by longitudinal shortening) by a compensation in LV wall thickness. Furthermore, in the presence of normal systolic function, LV hypertrophy resulted in enhanced EF. Of note, this relationship was not emulated when applied to SV; with end-diastolic wall thickness presenting no significant influence on resultant SV [30]. Although a similar increase in wall thickness was observed within the eccentric athletes (as compared to the concentric athletes), this increase in wall thickness was in combination with an increase in the LV cavity dimension and did not result in an enhanced EF; with EF comparable to normal geometry athletes. This “normalised” EF observed in the presence of an increased wall thickness and concomitant increase in LV cavity size was previously described [8]. The athletes in the current study exemplified this model; with concentric remodelling (increased wall thickness with no LV cavity enlargement) resulting in an enhanced EF, whereas eccentric remodelling (increased wall thickness with a concomitant LV cavity enlargement) eliciting a “normalisation” of EF to that comparable of normal geometry athletes; with all groups demonstrating a comparable SV.

### 4.2. Two-Dimensional Speckle Tracking Echocardiography

**Figure 6:**
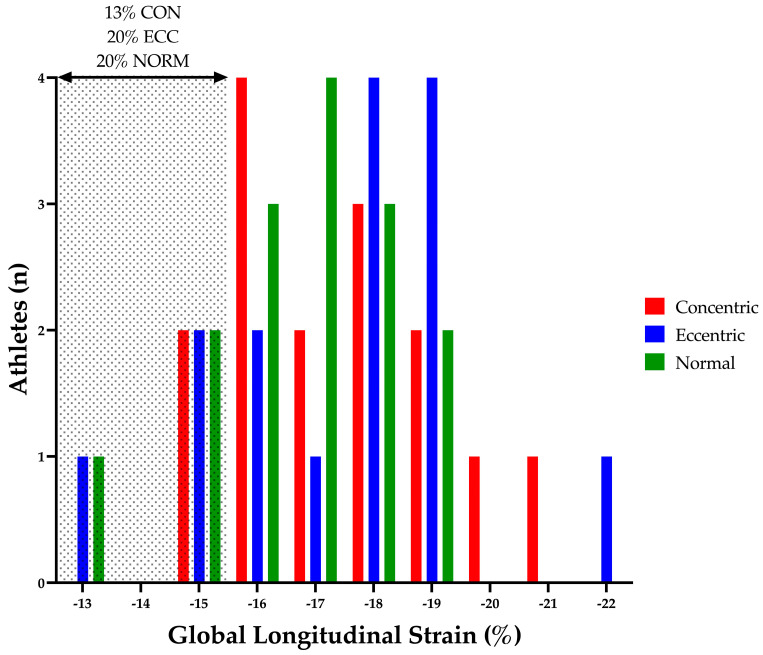
Left Ventricular Global Longitudinal Strain. Shaded area highlights athletes with a left ventricular global longitudinal strain outside of the normal limits (20% of NORM, 20% of ECC and 13% CON) [32]. CON, concentric remodelling/hypertrophy; ECC, eccentric hypertrophy; LV, left ventricular; NORM, normal geometry.

Peak myocardial strain and strain rates were similar in all athletic groups regardless of LV geometry. LV GLS is the most commonly cited mechanical parameter in clinical and AH studies [11]. Previous studies demonstrated equivocal finding with regard to GLS in athletes. Some studies reported lower values [31] as well as regional variance [8]. However, in the current study, the majority of athletes demonstrated a GLS within the recommended reference range (−16–22%; see Figure 6) [32]. Similar to these previous studies, a small percentage of athletes demonstrated reduced GLS outside of “normal limits” (i.e., <16%) (CON 13%; ECC 20%; NORM 20%; see Figure 6). Importantly, these athletes were equally distributed between LV geometry groups. Although impossible to definitively exclude underlying pathology in these athletes, the athlete ECGs did not present any abnormalities when using established international ECG criteria as a reference [25]; and therefore, we attribute these isolated low GLS values to represent the heterogenous nature of GLS in athletic populations.

Due to the absence of geometry-matched disease populations (hypertension, HTN; HCM; DCM), a direct comparison of the functional expression of physiological remodelling compared to pathological remodelling was impossible in the current study. However, previous studies demonstrated a reduced resting GLS in the presence of cardiovascular disease including HTN [33] HCM [20,21] and DCM [13] as compared with healthy athletes. Therefore, STE may provide a beneficial measure of LV systolic function in the differentiation of “grey zone” structural adaptation in athletes; with the identification of a reduction in myocardial strain, and more specifically, GLS, regardless of LV geometry, warranting further investigation. It is important to recognise that the current study compared resting myocardial strain. Interestingly, Schnell et al. [34] conducted a study comparing sedentary HCM patients, healthy sedentary controls, athletes with HCM and healthy athletic controls. Resting GLS was reported as reduced in the sedentary HCM group; with resting GLS comparable in the athletes with HCM and both control groups. However, exercise GLS allowed the differentiation of athletes with HCM and the athletic control group. Although exercise stress echocardiography was not conducted in the current study, previous studies suggested exercise STE and myocardial strain may provide additional aid in the differentiation of physiology from pathology in the presence of altered LV geometry.

### 4.3. Temporal Speckle Tracking Echocardiography

To the best of our knowledge, this is the first study to compare temporal strain curves in athletes with normal, concentric and eccentric remodelling. In the current study, peak myocardial strain and strain rates were similar between groups; however, temporal myocardial strain differences were present in concentric and eccentric LV remodelled athletes compared to normal geometry athletes (see Figure 4).

Concentric and eccentric remodelling demonstrated an association with higher mid systolic longitudinal and circumferential strain and strain rate. This suggests that remodelling in these athletes produced a potential enhancement in systolic function through earlier and increased strain and strain rate which was not represented by peak strain data alone. This demonstrates the potential complimentary benefit of temporal strain and strain rate analysis in the AH alongside peak myocardial strain assessment. In addition, eccentric remodelling was associated with enhanced early diastolic untwisting. During LV diastole, the ventricle rapidly untwists from the preceding end-systolic myocardial deformation generating a suction effect during early diastole to facilitate LV filling [35]. LV early diastolic flow constitutes the majority of LV diastolic filling, with late diastolic atrial systole contributing <25% of the LV stroke volume [36]. The rate of early diastolic filling is highly dependent on the rate of LV relaxation [37]. Although the current study does not allow for direct cross comparison to disease or control populations, enhanced untwisting was previously observed in athletes compared to HCM patients and sedentary controls [38]. The earlier and more marked early diastolic untwisting associated with eccentric remodelling suggests an enhanced LV compliance and early diastolic relaxation in this athletic group that is likely to become more relevant and beneficial during exercise where HR is increased and diastolic filling times decreased [39]. The combination of superior strain during early and mid-systole and enhanced untwisting highlights the potential positive functional adaptation generated by LV structural physiological remodelling in the AH.

### 4.4. Limitations

Although the current study utilised three groups of age, sex, ethnicity, training volume and sport-matched athletes, the individual group sample size was relatively small (*n* = 15) and may be slightly underpowered. An a priori sample size calculation based on previous athlete peak GLS demonstrated a sample size of 17 to determine 90% power for a −3% difference. All athletes included in the study were male and, therefore, these findings cannot be generalised to the female athletic population. Importantly, the relative prevalence of eccentric remodelling in the female athletic population is comparable to that of the male athletic population; however, concentric remodelling or hypertrophy is a rare presentation in female athletes [4].

The athletic population in the current study was divided into LV geometry groups as determined by LV mass index and RWT [24]. The majority of the population presented with normal to moderate structural adaptation in absolute LV wall thickness and internal chamber dimension (Figure 2 and Figure 3). As previously mentioned, LV myocardial strain is altered in marked LV pathological structural remodelling (HCM and DCM) [13,20,21]. Therefore, direct comparison through a cross sectional study utilising matched disease and athletic populations with more marked structural remodelling may be beneficial in establishing potential measures to aid differential diagnosis. Furthermore, cross sectional studies comparing negative or mild phenotype disease populations with matched athletic cohorts would be equally as important to identify early sub-clinical signs of disease.

In the current study, resting conventional 2D echocardiography and 2DSTE was utilised. Resting 3D echocardiography and 3DSTE as well as exercise echocardiography may provide beneficial insights into the association of LV geometry and myocardial strain in athletic populations.

## 5. Conclusions

Physiological concentric and eccentric remodelling in the AH is generally associated with normal LV function, with concentric remodelling associated with a higher EF compared to eccentric remodelling and those with normal geometry. Physiological concentric and eccentric remodelling in the AH had no effect on peak myocardial strain but superior deformation and untwisting was unmasked when assessing temporal myocardial strain.

## Figures and Tables

**Table 1:** Athlete Demographics.

	Concentric (n = 15; Median (Range))	Eccentric (n = 15; Median (Range))	Normal (n = 15; Median (Range))
Age (y)	24 (19–34)	24 (19–34)	25 (19–34)
Height (m)	1.82 (1.71–1.94)	1.82 (1.74–1.89)	1.82 (1.72–1.90)
Body Mass (kg)	76 (64–105)	76 (67–99)	76 (69–99)
Body Surface Area (m^2^)	1.99 (1.74–2.30)	1.96 (1.83–2.19)	1.95 (1.86–2.25)
Training Days per Week (days/week)	6 (5–7)	6 (5–7)	6 (5–7)
Training Hours per Week (hours/week)	20 (8–40)	15 (6–40)	17 (9–40)
Heart Rate (beats/min)	55 (40–67)	50 (39–65)	52 (38–95)

**Table 2:** Athlete Left Ventricular Structure and Function.

	Concentric (Median (Range))	Eccentric (Median (Range))	Normal (Median (Range))
LVIDd (mm)	50 (39–52) ‡†	57 (53–68) ‡*	53 (50–60) †*
LVIDdi (BSA; mm/m^2^)	25 (19–29) ‡†	29 (26–34) ‡*	27 (23–30) †*
LVIDdi^0.5^ (BSA^^0.5^; mm/m^2^0.5^)	34 (27–39) ‡†	40 (39–48) ‡*	38 (34–42) †*
LVIDs (mm)	33 (30–38) ‡†	37 (33–48) ‡	37 (33–40) †
LVIDsi (BSA; mm/m^2^)	17 (15–19) ‡†	19 (18–24) ‡	18 (16–21) †
LVIDsi^0.5^ (BSA^^0.5^; mm/m^2^0.5^)	24 (21–26) ‡†	27 (24–34) ‡	26 (24–28) †
LV Mass (g)	207 (105–233) ‡	226 (212–365) ‡*	191 (154–213) *
LV Massi (BSA; g/m^2^)	100 (50–127) ‡	116 (110–183) ‡*	99 (74–107) *
Mean Wall Thickness (mm)	10 (9–12) †	10 (9–11) *	9 (8–10) †*
Max Wall Thickness (mm)	11 (10–12) †	11 (9–12) *	10 (9–11) †*
RWT (ratio)	0.46 (0.42–0.51) ‡†	0.36 (0.27–0.40) ‡	0.36 (0.28–0.41) †
LVEDV (mL)	138 (79–201)	153 (125–198)	152 (125–192)
LVEDVi (BSA; mL/m^2^)	71 (43–99)	79 (57–99)	80 (56–100)
LVEDVi^1.5^ (BSA^^1.5^; mm/m^2^1.5^)	51 (32–69)	55 (39–72)	58 (37–72)
LVESV (mL)	52 (20–105)	68 (38–90)	65 (50–90)
LVESVi (BSA; mL/m^2^)	25 (11–51)	34 (20–48)	34 (24–46)
LVESVi^1.5^ (BSA^^1.5^; mm/m^2^1.5^)	17 (8–36) †	24 (15–35)	25 (16–33) †
Stroke Volume (mL)	87 (59–114)	94 (65–111)	90 (70–107)
Cardiac Output (L/min)	4.7 (2.8–6.8)	4.6 (2.9–5.6)	4.6 (3.3–7.0)
Ejection Fraction (%)	64 (48–78) †	57 (49–73)	56 (50–65) †

LVEDV, left ventricular end-diastolic volume; LVEDVi, left ventricular end-diastolic volume indexed; LVESV, left ventricular end-systolic volume; LVESVi, left ventricular end-systolic volume indexed; LVIDd, left ventricular end-diastolic internal dimension; LVIDdi, left ventricular end-diastolic internal dimension indexed; LVIDs, left ventricular end-systolic internal dimension; LVIDsi, left ventricular end-systolic internal dimension indexed; LV, left ventricle; LV Massi, left ventricular mass indexed; RWT, relative wall thickness. † Significant difference between concentric and normal ‡ Significant difference between concentric and eccentric * Significant difference between eccentric and normal.

**Table 3:** Athlete Left Ventricular Doppler and TDI.

	Concentric (Median (Range))	Eccentric (Median (Range))	Normal (Median (Range))
MV E (m/s)	0.77 (0.53–1.05)	0.74 (0.49–1.02)	0.73 (0.54–0.91)
MV A (m/s)	0.40 (0.20–0.58)	0.35 (0.23–0.55)	0.44 (0.24–0.63)
E:A (ratio)	1.88 (1.15–5.25)	2.06 (1.32–3.40)	1.86 (0.86–3.17)
Septal S’ (cm/s)	9 (7–11)	8 (7–10)	9 (8–11)
Septal E’ (cm/s)	11 (8–17)	11 (9–14)	12 (9–16)
Septal A’ (cm/s)	7 (5–10)	7 (5–10)	7 (5–9)
Lateral S’ (cm/s)	11 (8–17	11 (7–18)	12 (9–14)
Lateral E’ (cm/s)	19 (10–25)	17 (13–22)	19 (10–26)
Lateral A’ (cm/s)	7 (4–15)	7 (4–13)	6 (5–8)
Average S’ (cm/s)	10 (8–14)	10 (8–13)	10 (9–13)
Average E’ (cm/s)	16 (10–20)	14 (11–18)	16 (11–21)
Average A’ (cm/s)	7 (5–12)	7 (5–10)	7 (5–9)
Average E:E’ (ratio)	4.8 (3.4–8.1)	5.5 (3.6–6.2)	4.6 (3.6–7.3)
LVOT Velocity (m/s)	1.05 (0.67–1.68)	1.13 (0.84–1.5)	1.1 (0.78–1.22)
LVOT VTI (cm)	21.9 (14.6–32.1)	22.8 (18.0–32.2)	22.5 (16.2–25.9)
Aortic Velocity (m/s)	1.24 (1.15–1.78)	1.28 (1.04–1.73)	1.26 (1.04–1.45)

LVOT; left ventricular outflow tract; MV, mitral valve; VTI, velocity time integral.

**Table 4:** Left Ventricular Speckle Tracking Echocardiography.

	Concentric (Median (Range))	Eccentric (Median (Range))	Normal (Median (Range))
GLS (%)	−16.9 (−14.9–20.6)	−17.9 (−13.0–22.1)	−16.9 (−12.8–19.4)
GLSr S (s^−1^)	−0.91 (−0.77–1.04)	−0.85 (−0.70–1.07)	−0.80 (−0.73–1.09)
GLSr E (s^−1^)	1.32 (0.79–1.86)	1.21 (0.70–1.63)	1.29 (0.49–1.56)
GLSr A (s^−1^)	0.53 (0.37–0.66)	0.47 (0.28–0.95)	0.41 (0.28–0.71)
GCS (%)	−18.1 (−13.6–24.5)	−18.7 (−15.6–22.4)	−18.0 (−13.5–19.7)
GCSr S (s^−1^)	−1.02 (−0.80–1.41)	−1.09 (−0.87–1.26)	−0.97 (−0.73–1.22)
GCSr E (s^−1^)	1.45 (0.74–1.90)	1.43 (0.90–1.82)	1.28 (0.87–1.88)
GCSr A (s^−1^)	0.37 (0.10–0.61)	0.42 (0.18–0.75)	0.35 (0.22–0.47)
GRS (%)	42.2 (30.3–70.5)	50.0 (39.2–60.0)	40.6 (29.9–57.0)
GRSr S (s^−1^)	2.00 (1.45–2.77)	2.19 (1.62–2.89)	2.05 (1.47–3.03)
GRSr E (s^−1^)	−2.62 (−1.79–4.32)	−2.99 (−2.09–3.99)	−2.69 (−1.63–4.92)
GRSr A (s^−1^)	−1.08 (−0.64–1.83)	−1.18 (−0.62–2.21)	−1.14 (−0.67–2.24)
Twist (°)	14.9 (3.7–25.3)	12.5 (6.3–20.8)	13.2 (8.8–24.2)

A, late diastole; E, early diastole; GCS, global circumferential strain; GCSr, global circumferential strain rate; GLS, global longitudinal strain; GLSr, global longitudinal strain rate; GRS, global radial strain; GRSr, global radial strain rate; S, peak systole.

## Data Availability

The data presented in this study are available on request from the corresponding author. The data are not publicly available to maintain privacy.
